# Micronutrients in Maternal and Infant Health: Where We Are and Where We Should Go

**DOI:** 10.3390/nu15092192

**Published:** 2023-05-05

**Authors:** Yunxian Yu

**Affiliations:** Department of Epidemiology & Health Statistics, School of Public Health and Medicine, Zhejiang University, Hangzhou 310058, China; yunxianyu@zju.edu.cn

The first 1000 days of life are defined by the World Health Organization as a “window of opportunity” for a person’s growth and development, and nutrition is particularly important during this time window [1]. This Special Issue of *Nutrients*, “Micronutrients in Maternal and Infant Health: Where We Are and Where We Should Go”, focused on recent research about micronutrients in maternal and infant health. Micronutrients, which play a major role in the metabolism of cellular metabolism and the organ development of the fetus, are important for maintaining pregnancy, fetal growth, and infant health. This issue mainly includes four topics, which are vitamins, race elements, hemoglobin, single nucleotide polymorphisms (SNPs) of genes of the VitD metabolic pathway in maternal and infant health, and the association between micronutrients in maternal or cord blood.

In terms of vitamins, we mainly focused on vitamin D (VitD). VitD deficiency is common during pregnancy. Data from the Chinese Nutrition and Health Survey showed that the prevalence of VitD insufficiency or deficiency in pregnant women in China was about 96.0% [2]. Vitamin D deficiency has been linked to various complications during pregnancy and in infants. Hypertensive disorders of pregnancy (HDP), including gestational hypertension, preeclampsia, eclampsia, pregnancy complicated with chronic hypertension, and chronic hypertension complicated with preeclampsia [3], affect maternal and infant health in the near and long term [4]. Plasma 25(OH)D levels during pregnancy and SNPs in the genes of the VitD metabolic pathway are both important for HDP, as shown by Si et al. [5] In addition, Hu et al. [6] conducted a systematic review and meta-analysis, which indicated that VitD insufficiency or deficiency was associated with an increased risk of preeclampsia. Moreover, maintaining an adequate level of VitD is also beneficial to gestational diabetes (GDM) and thyroid function [7,8]. In addition, Lin et al. [9] suggested that the target group with both VitD deficiency and passive smoking should be paid more attention. Besides VitD, other vitamins are also receiving attention. Vitamin E is a lipid-soluble antioxidant that corrects the oxidative imbalance and protects tissue from damage. Vitamin B is a kind of water-soluble micronutrient, which plays a coenzyme role in many catabolic and anabolic enzyme reactions. Zhou et al. [10] and Li et al. [11] suggested that avoiding excessive vitamin E during pregnancy might be an effective measure to reduce GDM and instances of being large for the gestational age, and adherence to a diet pattern with high vitamin B during pregnancy was associated with higher birth weight and a lower risk of small-for-gestational-age (SGA).

In terms of trace elements, previous studies found that some trace elements participated in regulating and controlling enzyme activity and were involved in the synthesis of many lipids, nucleic acids, and proteins. Recent studies have reported that increased or decreased concentrations of some trace elements were associated with the risk of GDM. Wu et al. [12] also examined the association between maternal exposure to vanadium, chromium, manganese, cobalt, nickel, and selenium in early pregnancy and the risk of GDM, and found that vanadium was positively associated with the risk of GDM, while nickel was negatively associated. Dai et al. [13] reported that prenatal manganese exposure was negatively correlated with childhood physical development, which appeared to be most significant in the early stages. These studies emphasized the importance of detecting the concentrations of trace elements during pregnancy. In addition, dietary and dietary supplements are the main sources of zinc, copper, and selenium in pregnant women. Yang et al. [14] indicated that efforts to promote zinc and selenium intakes during pregnancy needed to be strengthened to reduce the incidence of congenital heart defects in the Chinese population.

Hemoglobin level is also associated with nutritional status and is regarded as a common and economical monitoring indicator for nutritional status assessment during pregnancy. However, the association between hemoglobin levels during pregnancy and the risk of adverse birth outcomes remains controversial. In addition, elevated maternal hemoglobin levels, compared with anemia, are often considered an indicator of good nutritional status and is not always given sufficient clinical attention. However, Liu et al. [15] and Peng et al. [16] evaluated the association between maternal hemoglobin levels and birth weight outcomes and indicated that both severe anemia and high hemoglobin (>130 g/L) should be paid attention to in the practice of maternal and infant health.

Finally, the concentration of micronutrients in human milk and the association between different micronutrients were also explored. Zhang et al. [17] conducted a meta-analysis and found that the level of vitamin A (VitA) in breast milk decreased with the course of breastfeeding, with little difference between China and other countries. These findings provided evidence for the improvement of VitA intake recommendations for exclusively breastfed infants under 6 months of age. In addition, Gang et al. [18] and Si et al. [19] evaluated the association between different micronutrients. Additionally, 25(OH)D levels in cord blood have been linked to diseases in children. Serum selenium and 25(OH)D have been reported in non-pregnant people, and Gang et al. [18] found a positive correlation between maternal urinary selenium levels and cord blood 25(OH)D levels. Si et al. [19] reported that VitD during pregnancy was positive for hemoglobin.

To summarize, micronutrient levels during pregnancy or in infants are essential for programming and the development of complications, which has important public health significance. The studies included in this Special Issue and other related research play important role in future evidence for developing guidelines and management of maternal and infant health through monitoring and supplementing micronutrients, especially for Chinese people. However, the combined effects of various micronutrients on maternal and infant health need to be further studied, and the relevant mechanisms also need to be further explored.
